# Analysis of corneal surface shape following overnight orthokeratology with different optical zone diameters

**DOI:** 10.3389/fmed.2024.1421361

**Published:** 2024-10-09

**Authors:** Minfeng Chen, Ronghan Zhang, Chengwei Zhu, Lulu Peng, Sijun Zhao, Xinjie Mao

**Affiliations:** Eye Hospital, Wenzhou Medical University, Wenzhou, Zhejiang, China

**Keywords:** orthokeratology, base curve, decentration, optical zone, myopia

## Abstract

**Purpose:**

This study analyzed the corneal surface shape following overnight orthokeratology with different optical zone diameters.

**Methods:**

A total of 82 eyes belonging to 41 myopic children who completed 1 month of the orthokeratology (ortho-k) lens wear at the Eye Hospital of Wenzhou Medical University from January 2022 to January 2023 were retrospectively analyzed. According to the size of the base curve (BC) of ortho-k lens, patients were divided into BC 5.0 and BC 6.0 groups. The changes in decentration distance and corneal refraction of the two groups after the ortho-k lens wear were analyzed. Independent sample *t*-tests were used to compare these two outcome measures between the two groups.

**Results:**

The decentration distance of BC 5.0 group (0.37 ± 0.19 mm) was significantly lower than that of BC 6.0 group (0.49 ± 0.25 mm, *t* = −2.330, *p* = 0.022). In the BC 5.0 group, the direction of decentration was superonasal in 3 cases, inferonasal in 2 cases, inferotemporal in 21 cases, and superotemporal in 6 cases. In the BC 6.0 group, the direction of decentration was superonasal in 2 cases, inferonasal in 2 cases, inferotemporal in 27 cases, and superotemporal in 19 cases. The optical zone area (8.19 ± 2.96 mm^2^) and reverse curve zone area (30.05 ± 6.74 mm^2^) in the BC 5.0 group were significantly lower than in the BC 6.0 group (10.42 ± 2.03 mm^2^, *t* = −4.043, *p* < 0.001; 38.21 ± 4.77 mm^2^, *t* = −6.422, *p* < 0.001). The change in the rate of refraction in the horizontal direction in BC 5.0 group were significant higher than in BC 6.0 group.

**Conclusion:**

Base curve 5.0 mm ortho-k lens is better positioned than BC 6.0 mm lens. A small BC ortho-k forms a smaller optical zone and reverse curve area, which might get a greater aiameter of alignment curve to facilitate positioning better than the traditional BC lens. In addition, a small BC lens increases positive refraction in the peripheral area, resulting in a greater negative pressure than the traditional BC lens.

## 1 Introduction

Among the many methods of controlling myopia, orthokeratology (ortho-k) represents a safe and effective method in slowing axial length (AL) elongation (1–5). However, the mechanism of ortho-k to slow the progression of myopia is still unknown. It is generally believed that ortho-k reshapes the anterior surface of cornea via inverse geometric design to flatten the central area of the cornea and form a steep peripheral area, resulting in peripheral defocus, which transforms peripheral retinal hyperopic defocus imaging into myopic defocus imaging for effective myopia control (1–8).

In order to further improve the effect of ortho-k on myopia control, the central base curve (BC) of the lens was reduced to obtain a small optical zone diameter lens, resulting in less AL elongation after wearing ortho-k lenses (9–14). Hu et al. (15) found that wearing ortho-k lens led to a greater change in corneal refraction of the smaller optical zone and reduced elongation of AL. Small BC ortho-k generates a smaller optical zone, which can increase myopic defocus in the peripheral area to achieve better myopia control (9). A reduced BC of the lens increases the need for lens fitting. The position of lens is worth further investigation.

The corneal topography generated by Medmont E-300 examination can be used to effectively evaluate the position and morphological characteristics of the optical zone after ortho-k wear (10–20).

Whether or not the lens positioning was centered is an important indicator during ortho-k treatment. The ideal lens fitting ensures a wider range of clear visual area and provides a safer corneal environment (3–14).

One month of ortho-k lens wear stabilized and maintained the direction of position (5, 14, 17–22). However, the optical zone was always inconsistent with the ideal location due to corneal asymmetry, eyelid tension, and lens movement (14–20). Even after basal lens fitting, the decentration of lens was inevitable after steady wear for 1 month. The expected best optical position was difficult to attain. This study provides a theoretical basis for the clinical fitting of small BC ortho-k lens by comparing the differences in corneal topography based on different optical zone diameters of ortho-k lens and analyses the decentration of small BC lens.

## 2 Materials and methods

### 2.1 Subjects

The study subjects included a total of 41 myopic children who completed the fitting and wore ortho-k lenses for more than a month in the Eye Hospital of Wenzhou Medical University from January 2022 to January 2023. The inclusion criteria were: (1) age, 8–11 years; (2) a diagnosis of myopia, best corrected visual acuity ≥5.0, spherical equivalent refraction (SER) ranging from −1.00 D to −6.00 D, astigmatism ≤1.50 D, corneal astigmatism < 2.00 D; (4) no history of ocular diseases, trauma, or systemic diseasesno other eye diseases; (5) no history of surgery or use of atropine or contact lenses to control myopia progression; (6) no systemic or ocular conditions that might affect vision. Usually the ortho-k of BC 6.0 (BC diameter 6.0 mm) size was used for the myopic children, but for the children with a fast progression of myopia (change of SER more than 0.75 D for 1 year), doctors suggested them could wear the ortho-k of BC 5.0 (BC diameter 5.0 mm) size to get a good effect on myopia control. Then this retrospective study collected the clinical data of two BC size (BC 5.0 group and BC 6.0) of ortho-k lenses available. Before preparing for ortho-k lens fitting, all subjects and their parents signed the informed consents following an detail discussion of the risk and benefits of wearing the ortho-k lenses. This study was performed in accordance with the tenets of the Declaration of Helsinki and was approved by the Wenzhou Medical University Eye Hospital ethics committee (2023-107-K-86).

### 2.2 Instrumentation

The ortho-k lenses used in this study included three-zoned reverse-geometry lenses (CRT, USA). Each subject underwent comprehensive baseline eye examination, including slit-lamp examination, testing for refraction, uncorrected visual acuity (UCVA), best-corrected visual acuity, AL (Zeiss IOL Master, Germany), corneal topography (E-300, Medmont International Pty. Ltd.), corneal endothelium microscopy, and intraocular pressure. All children were treated by doctors who had worked in the field of orthokeratology at the hospital for more than 10 years. Corneal topography was measured with a Medmont E-300 by a specialized technician within 1 h of removal of the ortho-k lenses. The topographic images used for analysis included each subject’s best-focus image (with an accuracy greater than 95%) from the four frames captured automatically. The doctor ordered the best lens for the subject based on each subject’s corneal topography and fitting evaluation of corneal fluorescein pattern analysis.

### 2.3 Measurements

#### 2.3.1 Corneal surface parameters

FK: flat radial refraction; SK: steep radial refraction; refractive power difference (ΔK: the difference of FK and SK), eccentricity on the flat meridian (Fe: flat radial eccentricity), FK and SK at 3 mm (from the vertex of the cornea), FK and SK at 5 mm (from the vertex of the cornea) and 7 mm (from the vertex of the cornea) were determined via corneal topography. SER, spherical refraction (SE) and astigmatism (As) were evaluated before wearing ortho-k lenses. The axial length (AL) was measured using IOL-Master 500, and UCVA was determined by re-examination after wearing the lens for 1 month.

#### 2.3.2 Measurement of areas and decentration distance

**FIGURE 1:**
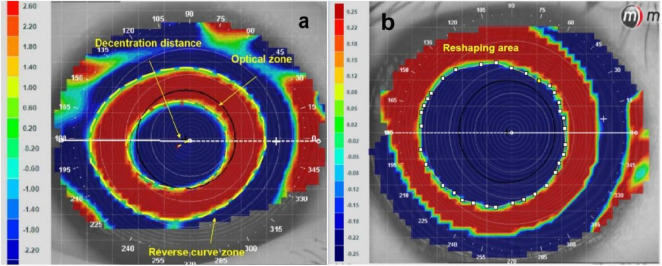
Analysis of the areas of reshaping zone and decentration distance via corneal topography. **(a)** Areas of reshaping zone in the tangential different topography. **(b)** Areas of reshaping area in the axial different topography.

Tangential difference topography and axial difference topography were determined at baseline and after 1 month of continuous ortho-k lens wear. According to the differences in topography, optical zone or reshaping area ranged from the corneal vertex to the point where the keratometry values changed within 1 D and less than two types of color (red and blue) in the refractive power parameters, as shown in Figure 1. Figure 1a shows the optical zone in tangential difference topography. Figure 1b presents the reshaping area in the axial difference topography. Importing the tangential difference topography into Matlab, 16 points were uniform drew in a clockwise fashion in the red-blue transition zone to delineate the margins of optical zone and then Matlab would position the center of optical zone. The decentration distance was measured from the corneal vertex and the center of optical zone (14–20). The reverse curve zone was blue-red transition zone, as shown in Figure 1a. Image J software was used to measure the area of optical zone, reverse curve zone, decentration distance, and reshaping area (14–20, 23).

#### 2.3.3 Optical zone parameters

**FIGURE 2:**
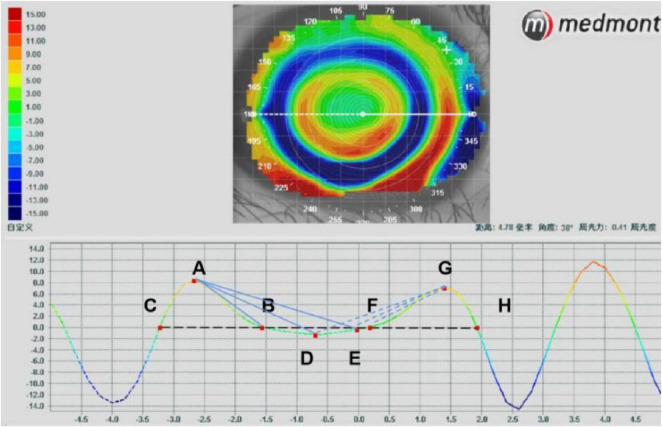
Analysis of the parameters of reshaping zone via corneal topography.

The parameters of optical zone were analyzed in the tangential difference topography (9, 24), as shown in Figure 2. In the horizontal direction, point A represents the highest point diopter of the peripheral steepened zone (PSZ) on the nasal side, while points B and C indicate the edge of the PSZ. Points G, F and H denote the same position of PSZ on the temporal side. Point D shows the lowest position of optical zone and point E indicates the position with 0 refraction. K_1_ refers to the average of AB and GH, which represent the slopes of change from peripheral peak (point A or G) to point zero at the optical zone edge (point B or H). K_2_ is the average of AD and GD, which represent the slopes of change from PSZ peak (point A or G) to the most negative power change (point D). K3 is the average of AE and GE, which represent the slopes of change from PSZ (point A or G) to the optical zone centre (point E). K_4_, K_5_ and K_6_ represent the K_1_, K_2_, K_3_ in the vertical direction, respectively. The formula for the calculation is as follows:


Diameterofopticalzone=F-xBx

DiameterofPSZ=[(B-xC)x+(H-xF)x]/2

K=1[A/y(B-xA)x+G/y(G-xF)x]/2

K=2[(A-yD)y/(D-xA)x+(G-yD)y/(G-xD)x]/2

K=3[(A-yE)y/(0-A)x+(G-yE)y/(G-x0)]/2

**FIGURE 3:**
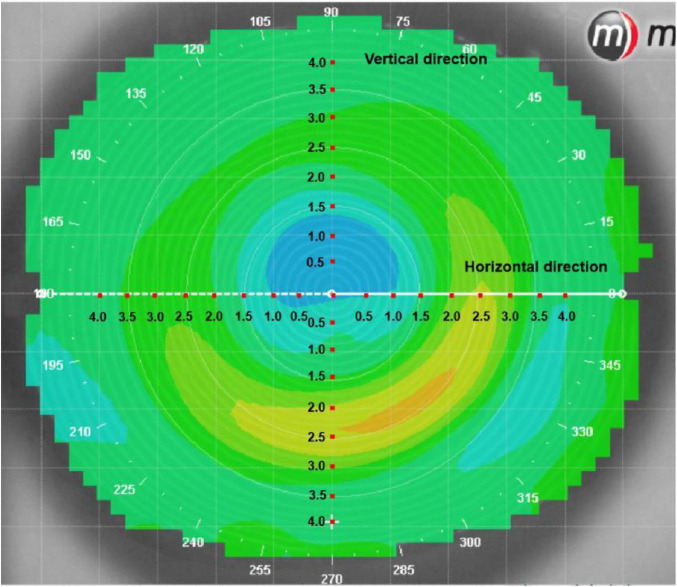
Analysis of the refraction of reshaping zone via corneal topography.

The changes in corneal refraction based on axial difference topography were determined by measuring the refraction along the horizontal and vertical meridians from the origin of corneal vertex (25, 26). The refraction was selected at an interval of 0.5 mm to analyze the changes in refraction in corneal topography with a diameter of 8 mm. The changes in refraction at 33 positions of the temporal, nasal inferior and superior cornea along the horizontal and vertical meridian were analyzed, as shown in Figure 3.

### 2.4 Statistical analysis

The data were processed using SPSS 24.0 for the Windows statistical software. The measurement data were expressed by *X* ± *s*. Data were first tested for normality using the Sample Shapiro-Wilk test. The independent sample T test was used to analyze the differences in group parameters. *P* < 0.05 was considered statistically significant.

## 3 Results

### 3.1 Basic information

Among 82 eyes of 41 myopic children, the average age was 9.10 ± 1.15 years. The average SER was −2.72 ± 1.03 D, and the average AL was 24.65 ± 0.68 mm. The BC 5.0 group comprised 16 cases (32 eyes) and the BC 6.0 group included 25 cases (50 eyes). No significant difference was found in basic parameters between the two groups, as shown in Table 1.

**TABLE 1:** Analysis of differences in baseline parameters of the two groups (BC 5.0 and BC 6.0).

Basic parameters	BC 5.0 (*n* = 32)	BC 6.0 (*n* = 50)	*t*	*P*
Age (year)	9.06 ± 1.27	9.12 ± 1.08	−0.219	0.827
SER (D)	−2.55 ± 1.03	−2.88 ± 0.96	1.490	0.140
SE (D)	−2.38 ± 0.96	−2.69 ± 0.96	1.422	0.159
As (D)	−0.34 ± 0.38	−0.39 ± 0.34	0.578	0.565
AL (mm)	24.66 ± 0.65	24.65 ± 0.71	0.029	0.978
UCVA	5.03 ± 0.16	4.95 ± 0.14	1.761	0.084
FK (D)	42.71 ± 0.97	42.77 ± 1.62	−0.215	0.831
SK (D)	43.70 ± 1.10	43.82 ± 2.53	−0.254	0.800
ΔK (D)	1.00 ± 0.40	1.05 ± 0.38	−0.544	0.588
Fe	0.63 ± 0.08	0.64 ± 1.08	−0.358	0.721
3 mm FK(D)	42.62 ± 0.98	42.60 ± 1.57	0.064	0.949
3 mm SK(D)	44.05 ± 1.20	44.17 ± 1.78	−0.338	0.736
5 mm FK (D)	42.26 ± 0.95	42.23 ± 1.59	0.101	0.920
5 mm SK (D)	43.69 ± 1.02	43.92 ± 1.69	−0.689	0.493
7 mm FK (D)	41.58 ± 0.92	41.43 ± 1.69	0.476	0.635
7 mm SK (D)	43.32 ± 0.84	43.54 ± 1.71	−0.673	0.503

SER, spherical equivalent refraction; SE, spherical refraction; As, astigmatism; AL, axial length; UCVA, uncorrected visual acuity after wearing OK lenses for 1 month; FK, flat radial refraction; SK, steep radial refraction; ΔK, difference between FK and SK; Fe, flat radial eccentricity; 3 mm, 3 mm from the vertex of the cornea; 5 mm, 5 mm from the vertex of the cornea; 7 mm, 7 mm from the vertex of the cornea.

### 3.2 Differences in areas and decentration distance between the two groups were analyzed after wearing for 1 month

**FIGURE 4:**
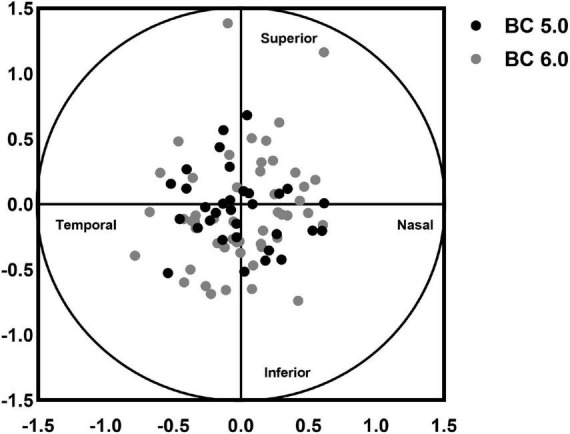
Analysis of the decentration direction of the two groups.

The decentration distance of BC 5.0 group (0.37 ± 0.19 mm) was significantly lower than that of BC 6.0 group (0.49 ± 0.25 mm, *t* = −2.330, *p* = 0.022). In the BC 5.0 group, the direction of decentration was superonasal in 3 cases, inferonasal in 2 cases, inferotemporal in 21 cases, and superotemporal in 6 cases. In the BC 6.0 group, the direction of decentration was superonasal in 2 cases, inferonasal in 2 cases, inferotemporal in 27 cases, and superotemporal in 19 cases, as shown in Figure 4. The optical zone area (8.19 ± 2.96 mm^2^) and the reverse curve zone area (30.05 ± 6.74 mm^2^) in the BC 5.0 group were significantly lower than in the BC 6.0 group (10.42 ± 2.03 mm^2^, *t* = −4.043, *p* < 0.001; 38.21 ± 4.77 mm^2^, *t* = −6.422, *p* < 0.001). The reshaping area in the BC 5.0 group (15.95 ± 4.40 mm^2^) was also significantly smaller than in the BC 6.0 group (23.21 ± 3.63 mm^2^, *t* = −8.121, *p* < 0.001). However, no significant difference was found in the ratio of optical zone between BC 5.0 and BC 6.0 groups, as shown in Table 2.

**TABLE 2:** Analysis of differences in corneal reshaping between the two groups (BC 5.0 and BC 6.0).

Groups	BC 5.0 (*n* = 32)	BC 6.0 (*n* = 50)	*t*	*P*
Optical zone (mm^2^)	8.19 ± 2.96	10.42 ± 2.03	−4.043	<0.001
Reverse curve zone (mm^2^)	30.05 ± 6.74	38.21 ± 4.77	−6.422	<0.001
Ratio of optical zone (%)	26.99 ± 0.06	27.26 ± 0.04	−0.236	0.814
Reshaping area (mm^2^)	15.95 ± 4.40	23.21 ± 3.63	−8.121	<0.001
Decentration distance (mm)	0.37 ± 0.19	0.49 ± 0.25	−2.330	0.022

### 3.3 Differences in optical zone parameters of the two groups

The diameters of optical zone (2.98 ± 0.48 mm) and PSZ (1.51 ± 0.22 mm) in the BC 5.0 group were significantly smaller than that in the BC 6.0 group (3.53 ± 0.54 mm, *t* = −4.622, *p* < 0.001; 1.69 ± 0.19 mm, *t* = −3.778, *p* < 0.001) both in horizontal and vertical directions. In the horizontal direction, the slopes of K_1_ (BC 5.0: 8.21 ± 3.55, BC 6.0: 6.41 ± 2.91, *t* = 2.480, *p* = 0.015), K_2_ (BC 5.0: 4.04 ± 1.24, BC 6.0: 3.26 ± 0.95, *t* = 3.207, *p* = 0.002) and K_3_ (BC 5.0: 4.28 ± 1.57, BC 6.0: 3.37 ± 1.04, *t* = 3.131, *p* = 0.002) of the BC 5.0 group were all greater than in the BC 6.0 group. However, no statistically significant differences were found between the two groups in vertical direction. As shown in Table 3.

**TABLE 3:** Analysis of the differences in reshaping situation between the two groups (BC 5.0 and BC 6.0).

Groups	BC 5.0 (*n* = 32)	BC 6.0 (*n* = 50)	*T*	*P*
**Horizontal direction**				
Diameter of OZ (mm)	2.98 ± 0.48	3.53 ± 0.54	−4.622	<0.001
Diameter of PSZ (mm)	1.51 ± 0.22	1.69 ± 0.19	−3.778	<0.001
K_1_	8.21 ± 3.55	6.41 ± 2.91	2.480	0.015
K_2_	4.04 ± 1.24	3.26 ± 0.95	3.207	0.002
K_3_	4.28 ± 1.57	3.37 ± 1.04	3.131	0.002
**Vertical direction**				
Diameter of OZ (mm)	2.96 ± 0.55	3.41 ± 0.44	−4.115	<0.001
Diameter of PSZ (mm)	1.41 ± 0.25	1.57 ± 0.36	−2.162	0.034
K_5_	10.46 ± 3.48	9.52 ± 4.49	0.994	0.323
K_6_	4.50 ± 1.17	4.18 ± 1.45	1.045	0.299
K_7_	5.70 ± 1.82	5.15 ± 2.06	1.223	0.225

K_1_ denotes the average of AB and GH, which represent the slopes of change from peripheral peak (point A or G) to point zero at the optical zone edge (point B or H); K_2_ denotes the average of AD and GD, which represent the slopes of change from PSZ peak (point A or G) to the most negative power change (point D); and K3 refers to the average of AE and GE, which represent the slopes of change from PSZ (point A or G) to the optical zone centre (point E). K_4_, K_5_ and K_6_ represent K_1_, K_2_, K_3_ in vertical direction, respectively.

### 3.4 Changes in corneal refraction between the two groups

**FIGURE 5:**
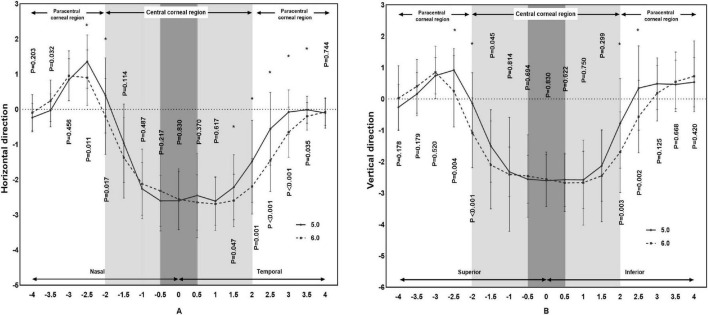
Comparison of differences in refraction based on corneal topography of subjects wearing orthokeratology lenses in the two groups.

The changes in refraction of the BC 5.0 group were significantly greater than in the BC 6.0 group in the horizontal direction of 2.0 mm (*P* = 0.017), 2.5 mm (*P* = 0.011) on the nasal side and 1.5 mm (*P* = 0.047), 2.0 mm (*P* = 0.001), 2.5 mm (*P* < 0.001), 3.0 mm (*P* < 0.001), 3.5 mm (*P* = 0.035) on the temporal side. The changes in refraction of the BC 5.0 group were significantly greater than in the BC 6.0 group along the vertical direction of 2.0 mm (*P* < 0.001), 2.5 mm (*P* = 0.004) on the superior side and 2.0 mm (*P* = 0.003), 2.5 mm (*P* = 0.002) on the inferior side, as shown in Figure 5.

## 4 Discussion

Orthokeratology is an effective method to slow elongation in AL (1–5). Guo et al. (11) confirmed that small BC design ortho-k lenses with reduced BC were more effective in controlling myopia than traditional ortho-k lenses with great BC design. After reducing the BC, the positioning of small BC lens requires attention. Compared with the traditional ortho-k design, this study found that the small BC design lens formed a smaller optical zone after wearing. The diameters of the optical zone on the horizontal or the vertical meridian were significantly smaller than in the traditional lens. These results were consistent with the results reported by Gifford et al. (10).

Although the small BC lens formed a smaller optical zone after wearing, it ensured a better position with a lower decentration distance than the traditional BC lens. According to Wang and Yamg (27) and Gu et al. (28), the decentration distance was 0.51 ± 0.23 mm and 0.73 ± 0.25 mm, respectively. In the study of Chen et al. (14) the decentration distance was 0.64 ± 0.38 mm and 0.68 ± 0.32 mm at 3 and 24 months, respectively, after wearing the lens. The decentration distances measured by Yang et al. (22) at 1, 3, and 6 months after lens wear were 0.57 ± 0.41 mm, 0.55 ± 0.48 mm, and 0.59 ± 0.39 mm, respectively. In this study the decentration distance measured in the BC 6.0 group was 0.49 ± 0.25 mm, which was similar to the results of these studies. The decentration distance in the BC 5.0 group was 0.37 ± 0.19 mm, which was less than in the BC 6.0 group and these studies (14, 22, 27, 28). The main direction of decentration in both groups was inferotemporal, which was consistent with previous studies (14, 22, 27, 28). Wearing small BC ortho-k lens might get a larger diameter of alignment curve for better positioning than the larger BC lens.

Remy (9) reported that the horizontal diameter of the optical zone was 3.84 ± 0.38 mm and the vertical diameter was 3.81 ± 0.30 mm, and the diameter of PSZ in different directions ranged from 1.58 ± 0.18 to 1.70 ± 0.23 mm after lens wear. In this study, the horizontal diameter of optical zone (3.53 ± 0.54 mm) and PSZ (1.69 ± 0. 19 mm) as well as the vertical diameter of optical zone (3.41 ± 0.44 mm) and PSZ (1.57 ± 0.36 mm) in the BC 6.0 group were consistent with the results reported by Remy. Both optical zone and PSZ diameters in the BC 5.0 group were all lower than in the BC 6.0 group and the results of Remy study. Further, the small BC lens formed a smaller reverse curve zone than the tradition BC lens. However, the ratio of optical zone in the BC 5.0 group was consistent with that of the BC 6.0 group, suggesting that the small BC lens formed a smaller optical zone and a peripheral defocus zone. However, the ratio of optical zone might not be affected by the differences in lens design.

By reshaping the anterior surface of the cornea with a special reverse geometric design (1–8), ortho-k lenses can be used to change the peripheral retinal hyperopic defocus imaging to myopic defocus for effective myopia control. Hu’s study (15) reported that the rapid changes in corneal refraction in the optical zone slowed the AL elongation after lens wear. Chen et al. (29) found that additional areas of positive refraction in the central corneal zone were more effective for myopia control. These indicated that increased and rapid changes in corneal refraction were related to myopia control. Zhang et al. (12) reported that the changes in corneal refraction of the BC 5.0 mm aspheric lens were greater than in BC 6.0 mm and BC 6.2 mm lenses. The higher number of positive refraction zones in the BC 5.0 lens may be more effective in myopia control compared with the BC 6.0 lens (12, 15). Although in this study the BC 5.0 lens was spherical, it also increased the number of positive refractions in the peripheral zone compared with the BC 6.0 lens. These more positive refractions might increased more myopic defocus in the peripheral area to achieve better myopia control.

The concern is the issue of vision after wearing small ortho-k lens. A smaller optical area may lead to poorer vision. No statistically significant difference in UCVA was found between the two groups after wearing the ortho-k lens for 1 month in this study. Carracedo et al. (20) found that the fourth-order spherical aberration increased after wearing the BC 5.0 lens, which might affect only the contrast sensitivity. No significant differences in visual sensitivity and subjective visual acuity were found between BC 5.0 lens and BC 6.0 lens in Carracedo’s study. Gifford et al. (10) found that the optical zone was reduced after wearing the aspheric lens, which did not affect the acuity after lens wear. Although the refraction of peripheral positive zone in BC 5.0 lens was greater than in BC 6.0 lens, the refraction of central zone in BC 5.0 lens was consistent with that of the BC 6.0 lens, resulting in similar uncorrected visual acuity in the two groups. Might be this range of optical zone in BC 5.0 group could afford a acceptable UCVA. However, more research is needed to analyze the what is the require size of optical zone can achieve ideal vision.

The study has some limitations. First, the calculation of the sample size suggests the need for more than 20 cases in the two groups. However, using the small sample size, we could only analyze the binocular conditions after ortho-k lens wear. Second, although previous studies reported that the small BC lens had a significant effect on myopia control than the traditional BC lens, a few subjects wore the lens only for a short time, which hindered the analysis of myopia progression. Therefore, this study only analyzed lens fitting, which could not analyze the relationship with myopia control, suggesting the need for further investigation.

## 5 Conclusion

Small BC ortho-k lenses might provide a better position with less decentration distance than traditional BC lens. Wearing small BC ortho-k lens can lead to the formation of smaller optical zone and a reverse curve zone, which might get a larger diameter of alignment curve for better positioning than the larger BC lens. In addition, a smaller BC lens can yield additional positive refraction in the peripheral area, which might generate a greater negative pressure for better positioning than the large BC lens.

## Data Availability

The data analyzed in this study is subject to the following licenses/restrictions: the raw data supporting the conclusions of this article will be made available by the authors, without undue reservation. Requests to access these datasets should be directed to Minfeng Chen, 2040685010@qq.com.
